# Multiplexed Knockouts in the Model Diatom *Phaeodactylum* by Episomal Delivery of a Selectable Cas9

**DOI:** 10.3389/fmicb.2020.00005

**Published:** 2020-01-28

**Authors:** Mark Andrew Moosburner, Pardis Gholami, James K. McCarthy, Maxine Tan, Vincent A. Bielinski, Andrew E. Allen

**Affiliations:** ^1^Integrative Oceanography Division, Scripps Institution of Oceanography, University of California, San Diego, La Jolla, CA, United States; ^2^J. Craig Venter Institute, La Jolla, CA, United States

**Keywords:** diatom, gene editing, CRISPR-Cas9, conjugation, multiplex, bioengineering, mutant, *Phaeodactylum*

## Abstract

Marine diatoms are eukaryotic microalgae that play significant ecological and biogeochemical roles in oceans. They also have significant potential as organismal platforms for exploitation to address biotechnological and industrial goals. In order to address both modes of research, sophisticated molecular and genetic tools are required. We presented here new and improved methodologies for introducing CRISPR-Cas9 to the model diatom *Phaeodactylum tricornutum* cells and a streamlined protocol for genotyping mutant cell lines with previously unknown phenotypes. First, bacterial-conjugation was optimized for the delivery of Cas9 by transcriptionally fusing Cas9 to a selectable marker by the 2A peptide. An episome cloning strategy using both negative and positive selection was developed to streamline CRISPR-episome assembly. Next, cell line picking and genotyping strategies, that utilize manual sequencing curation, TIDE sequencing analysis, and a T7 endonuclease assay, were developed to shorten the time required to generate mutants. Following this new experimental pipeline, both single-gene and two-gene knockout cell lines were generated at mutagenesis efficiencies of 48% and 25%, respectively. Lastly, a protocol for precise gene insertions via CRISPR-Cas9 targeting was developed using particle-bombardment transformation methods. Overall, the novel Cas9 episome design and improved genotyping methods presented here allow for quick and easy genotyping and isolation of *Phaeodactylum* mutant cell lines (less than 3 weeks) without relying on a known phenotype to screen for mutants.

## Introduction

Marine diatoms are of biotechnological significance for their capacity to assimilate large amounts of carbon and nitrogen, divide rapidly (Cermeño et al., 2011), thrive at high cell densities, and fix carbon via photosynthesis. Diatoms shunt nutrients toward metabolic processes that produce industrially valuable products such as biodiesel precursors (triglycerides a.k.a. TAGs), polyunsaturated fatty acids (PUFAs), and photosynthetic pigments (Pulz and Gross, 2004; Vinayak et al., 2015). Regardless of their innate ability to produce these high-valued molecules (HVMs), increasing production beyond their native capabilities is desirable for reducing production costs, and achieving sustainability goals in industrial agriculture applications (Gao, 2018). Utilization of genetic engineering technologies could shift metabolic outputs of marine diatoms toward these HVMs and improve commercial production where algae have been suggested as biological platforms for industrial feedstock and nutraceutical production (Vinayak et al., 2015). Nevertheless, the development and optimization of such genetic engineering technologies is necessary before they can be safely, easily, and robustly employed.

Genetic engineering technologies, particularly the versatile CRISPR-Cas9 toolbox, have been used for numerous applications in a vast array of organisms (Mali et al., 2013b; Sanders and Joung, 2014; Wang et al., 2016). CRISPR (clustered regularly interspaced short palindromic repeats), a foreign nucleic acid immunity system, is encoded in the genomes of most bacteria and some archaeal species (Deltcheva et al., 2011). The type-II CRISPR system from the bacterium *Streptococcus pyogenes* was first adapted for gene editing using the Cas9 enzyme, which induces blunt-ended double-stranded breaks (DSBs) when guided to a genetic target by a chimeric RNA molecule of the tracr-RNA and cr-RNA called a single-guided RNA (sgRNA) (Cong et al., 2013; Jinek et al., 2013; Mali et al., 2013a). Together Cas9 and an sgRNA form a ribo-endonuclease complex *in vivo* in many cell types to edit or mutate genomic targets. In the bacterial immunity system, the sgRNA targeting sequence, a string of ∼20 nucleotides (nt), preserves the sequence of a previous immune response, called spacer acquisition (McGinn and Marraffini, 2018); whereas in gene editing applications the nucleotide sequence of 20-nt spacer is designed by the researcher to target a specific gene/target, followed by interrogation and cleavage of a nucleic acid target by the Cas9 endonuclease. The sgRNA guides Cas9 to the target by forming complementary base pairing with the 20 nt at the 5′ end of the sgRNA, called the spacer. Once a DSB is induced, the blunt ended breaks are repaired by one of two native DNA repair mechanisms: homology-directed repair (HDR) or non-homologous end joining (NHEJ). HDR relies on a homologous donor to repair the DSB, typically a sister chromosome in eukaryotes, by homologous recombination (LaFave and Sekelsky, 2009). Endogenous HDR can be hijacked in order to introduce, at the DSB, an exogenous donor sequence, containing a user-designed mutation that will result in an edited target locus. For NHEJ, ligation of the blunt ends repairs the DSB, however, this process is error-prone and leads to random insertions or deletions of nucleotides of unpredictable sizes called indels (Davis and Chen, 2013).

CRISPR-Cas9 has been employed in both prokaryotic and eukaryotic organisms but eukaryotes pose particular challenges for CRISPR-Cas9 experiments as most have multiple copies of their chromosomes. Multiple copies of chromosomes provide two or more target loci for Cas9. For instance, in a diploid organism, two individual DNA repair events must occur to produce homozygous or heterozygous mutations at the target loci. For NHEJ-mediated mutagenesis (where no homologous donor is introduced), a heterozygous genotype arises after two separate Cas9 cleavage events and NHEJ-mediated repair that results in two distinct indels at the target loci. A homozygous genotype can also arise when one indel-containing target locus is used as a homologous donor for the second target loci (located on the sister chromosome of the first target), thereby producing an identical indel mutation. The unpredictability of NHEJ-mediated mutagenesis in diploid organisms therefore requires highly sensitive genotyping methodologies. Many genotyping methods have been used to genotype diploid organisms; among the most common are high-resolution melt curve analysis (HRMC) (Nymark et al., 2016), T7 endonuclease I assay (Guschin et al., 2010; Cho et al., 2013), high-throughput next generation sequencing (Zhou et al., 2014), and manual curation of sequencing reads.

Marine diatom species have been subjected to CRISPR-Cas9 mutagenesis including the model strains: the pennate, *Phaeodactylum tricornutum* (Nymark et al., 2016; Serif et al., 2018; Sharma et al., 2018; Slattery et al., 2018; Stukenberg et al., 2018) and the centric *Thalassiosira pseudonana* (Hopes et al., 2016). To date, NHEJ-mediated mutagenesis has been observed only in *Phaeodactylum* (Nymark et al., 2016; Serif et al., 2018; Slattery et al., 2018) by using one of two genetic transformation methods, micro-particle bombardment or bacterial conjugation, to introduce CRISPR-Cas9. Despite the successes of utilizing CRISPR-Cas9 in generating mutant diatom cell lines, genotyping the generated cell lines has been difficult. *Phaeodactylum*, a diploid organism, requires high-sensitivity screening methods to genotype multiple cell lines in a high-throughput manner. Genotyping methods such as high-throughput melt curve (HRMC) analysis and the T7 endonuclease I assay are commonly used to evaluate mutant genotypes in multi-ploidy organisms. To date, HRMC analysis and CRISPR Activity Factor (CAF) analysis have been used for *Phaeodactylum* genotyping experiments (Nymark et al., 2016; Sharma et al., 2018; Stukenberg et al., 2018). Similarly to CAF, TIDE (Tracking Indels by DEconvolution) sequencing analysis, an open-source software package, deconvolves raw sequencing reads to reveal indels in diploid or multiploidy organisms (Brinkman et al., 2014; Serif et al., 2018). Allele-specific PCR amplification has been used for genotyping *Phaeodactylum* mutants without the need for subcloning (Serif et al., 2018).

Here, TIDE sequencing analysis and manual sequencing curation is used to streamline the genotyping pipeline from colony formation to mutant cell line validation. Both single-gene and two-gene mutant cell lines were produced utilizing conjugation to introduce a selectable-Cas9 and either one or two sgRNAs to *Phaedocatylum.* Also, the T7 endonuclease I assay has also been adapted to quantify the mutagenesis efficiency of individual sgRNAs throughout a population of transformed *Phaeodactylum* prior to genotyping individual colonies.

A new selectable-Cas9 episome design was used that permits antibiotic selection for the Cas9 gene with the goal of both increasing mutagenesis efficiency and potentially avoiding mixed genotypes forming in generated colonies after conjugation. The P2A self-cleaving peptide was used to transcriptionally fuse Cas9 and the *Phaeodactylum* antibiotic resistant gene, *sh ble* (Kim et al., 2011). By doing so, Cas9 and *sh ble* are co-transcribed under the same promoter followed by cleavage of the translated products, essentially selecting for Cas9 via antibiotic treatment. Lastly, a robust golden-gate assembly based method was developed to visualized positive and negative *E. Coli* clones that harbor ready-to-conjugate episomes. Overall, the methods presented here allow for easy Cas9-sgRNA episome assembly and streamlined genotyping methodology and isolation of *Phaeodactylum* mutant cell lines in less than 3 weeks without relying on a known phenotype to screen for mutants.

## Materials and Methods

### Cell Culturing

A reference strain of *Phaeodactylum* (CCAP-1055/1) was used in all experiments. *Phaeodactylum* was grown at 18°C under white fluorescent lights (50 uE m^–2^ s^–1^) and subjected to a diel growth cycle (14 h:10 h; light:dark). Culture medium was artificial sea water (ASW) supplemented with trace metals, essential vitamins, 55 uM of NaPO_4_, and 880 uM of the appropriate nitrogen source (NaNO_3_, NH_4_Cl, or Urea). Cultures, when not used in growth assays, were maintained with chloramphenicol antibiotic (10 mg/L) to keep cultures bacteria-free. Mutant *Phaeodactylum* strains were supplemented with either phleomycin (50 mg/L), zeocin (50 mg/L), or nourseothricin (200 mg/L).

*Escherichia coli* (TOP-10, Life Technologies, Carlsbad, CA) was used for all molecular cloning purposes. The cultures were grown on Luria-Bertani broth or agar and supplemented with the following antibiotics when necessary: ampicillin (100 mg/L), carbicillin (100 mg/L), tetracyclin (10 mg/L), gentamicin (20 mg/L), zeocin (25 mg/L).

### Molecular Biology

Plasmid construction was performed with reference to Sambrook and Russell (2001) unless stated otherwise. Multiple DNA polymerases were used for cloning purposes including Phusion High-Fidelity DNA polymerase (Thermo Fisher Scientific, Waltham, MA, United States), AccuPrime Taq High-Fidelity DNA polymerase (Thermo Fisher Scientific), OneTaq 2X Master Mix with standard buffer (New England Biolabs, Ipswich, MA, United States), and Phire Plant Direct PCR Master Mix (Thermo Fisher Scientific). Enzymatic components to make a 2X GA master mix that included Phusion HF DNA polymerase, T5 exonuclease (Thermo Fisher Scientific), and DNA Taq Ligase (Thermo Fisher Scientific) were purchased separately and mixed in lab. Enzymatic components for Golden-Gate cloning were also purchased separately but only mixed on the day of cloning. They included the type-II restriction enzymes *Bsa*I*-HF* and *Bbs*I (New England Biolabs) and a T4 DNA Ligase (Thermo Fisher Scientific).

Primer sequences can be found in the Supplementary Material.

#### CRISPR/Cas9 Genetic Components

A human-codon optimized Cas9 open reading frame (ORF) containing three nucleus-localization signals (NLS) was produced by the Feng Zhang laboratory (Broad Institute, Cambridge, MA, United States) and purchased from addgene.com [pSpCas9(BB)-2A-Puro (PX459) V2.0, ID: 62988, Ran et al., 2013]. The Cas9 ORF was cloned into a pUC19 backbone using GA cloning (Gibson et al., 2009) using primers Cas9-2A-shble_1 and P2A_1 (Supplementary Material). For constitutive expression of Cas9, the FcpB/FcpA (Fucoxanthin chlorophyll a/c promoter B/Fucoxanthin chlorophyll a/c terminator A) (Bailleul et al., 2010) promoter/terminator pair were chosen (FcpB-Cas9).

The HDR-donor plasmid used in Weyman et al. (2014) for TALEN mutagenesis was adapted to a HDR-donor plasmid for targeting *nitrate reductase* (pKO-NR). pKO-*NR* contained 1 kb homology arms flanking an expression cassette coding for the *sh ble* gene that conferred resistance to the antibiotic phleomycin. The cassette inserted both a sh ble cassette and a STOP codon upstream of the sh ble promoter (Fucoxanthin chlorophyll a/c binding promoter F, FcpF). This was in frame with the targeted coding gene. The primers and plasmid sequence information can be found in Weyman et al. (2014).

The sRNAi promoter (chr2: 28,038-29,083) and terminator (chr2: 29,124-29,424) was amplified from *Phaeodactylum* genomic DNA (promoter primers = sRNAi1_1 and sRNAi1_2; terminator primers = sRNAi_3 and sRNAi_4). For Golden Gate cloning, the sRNAi terminator was shortened by PCR to 112 bp to eliminate an internal *Bsa*I restriction site. The sRNAi promoter and terminator was cloned from *Phaeodactylum* genomic DNA at the locus of the most highly expressed small, non-coding, intergenic RNA called sRNAi (Rogato et al., 2014; Bielinski et al., 2017). The sequences for the sRNAi promoter and terminator can be found in the Supplementary Material.

The sgRNA backbone, which consists of the genetic fusion of the crRNA and tracrRNA (Jinek et al., 2012), was amplified from the same Addgene plasmid that contained Cas9 (primers = sRNAgU1_F and sRNAgU1_R). An sgRNA expression cassette for *Phaeodactylum* (sRNAi*_gBbsI*) was constructed using a 4-piece GA approach into an *Xba*I-digested pUC19 plasmid containing a bacterial expression cassette for the *bla* gene conferring resistance to ampicillin/carbicillin. The resulting plasmid was then PCR amplified at the 3′ end of the sRNAi and the 5′ end of the sgRNA backbone (primers = gRNA_1 and gRNA_2). This amplicon served as a linearized vector for a 2-piece GA procedure for a spacer insert that contains two flanking *Bbs*I restriction sites (Supplementary Figure S2). The 100 bp GA insert was constructed by ligating two 60 bp oligos with homology to the sRNAi promoter and sgRNA backbone. The resulting plasmid, *sRNAi_g2XBbsI*, was used for *Bbs*I-based GG cloning of CRISPR spacers (Supplementary Figure S2B).

For all Cas9 target genes, spacer sequences were obtained for *nitrate reductase (Phatr3_J54983)*, *glutamine synthetase II* (*GS-2*) (*Gene ID: Phatr3_J51092*), and *chloroplastic glutamate synthase* (*cGOGAT*) (*Gene ID: Phatr3_J24739*) using the Phyto CRISP-Ex online package (Rastogi et al., 2016) that passed the built-in criteria of minimizing off-target Cas9 activity.

A GG cloning protocol was modified from the sgRNA expression cassette supplied by Feng Zhang laboratory. In place of the *Bbs*I-containing spacer sequence within the sgRNA expression plasmid, a LacZ bacterial expression cassette with flanking *Bbs*I sites was cloned (primers = LacZ_GG_1 and LacZ_GG_2) into the sRNAi expression cassette using GA. The Pt sgRNA expression plasmid previously produced (sRNAi_*gBbsI*) was PCR amplified (primers = LacZ_GG_3 and LacZ_GG_4) and the amplicon was gel extracted and used as a GA vector. The sgRNA cloning vector (sRNAi_gLacZ) was the assembled plasmid. This plasmid was used in all future sgRNA expression cassette cloning and assembly experiments as a cloning vector (Supplementary Figure S1B). An appropriate GG insert for sRNAi_gLacZ was assembled by ligating two 56-nt oligos together that are complimentary to each other. The oligo primer sequences for all sgRNA target sequences can be found in Supplementary Material.

Detailed protocols for spacer cloning into the sRNAi_gLacZ plasmid (name GG1 cloning) are available at protocols.io (doi: 10.17504/protocols.io.4abgsan).

#### Cas9-Episomal Plasmid Cloning

The *Phaeodactylum* episomal plasmid pPtPBR1 was obtained from R. Diner (Diner et al., 2016). First, one *Bsa*I restriction site within the *bla* coding region of pPtPBR1 was removed by PCR amplification (primers = pBR322-Amp_1 and pBR322-Amp_2) and reassembly to produce plasmid pPtPBR1 (*Bsa*I-). pPtPBR1 was then used as a template for GA cloning of a Cas9 ORF and a GG cloning *Bsa*I acceptor site for sgRNA expression cassette cloning (Supplementary Figure S2). Cas9 was PCR amplified twice using one forward primer (Cas9-2A-sh ble_1) and two reverse primers (P2A_1 and P2A_2) so to add GA homology to the FcpF promoter (5′ of Cas9) and the sh ble ORF (3′ of Cas9). The double PCR reaction added a GSG-2A peptide DNA coding sequence to transcriptionally fuse hCas9 and sh ble (Supplementary Figure S2, GSG-P2A nucleotide sequence is in Supplementary Material). Next, a red-fluorescent protein bacterial expression cassette (mRFP) was obtained and PCR amplified (primers = Cas9-2A_sh ble-RFP_1 and 2) to produce GG acceptor sites. The remaining two GA pieces were PCR amplified from pPtPBR1 (*Bsa*I-) to insert two GG cloning *Bsa*I sites between the *bla* and *tet* expression cassettes and flanking mRFP, one using primers Cas9-2A-sh ble_2 and 3 (5′ – sh ble – Pt_Centromere – pMB1 – bla – 3′) and two using primer Cas9-2A-sh ble_6 and 7 (5′ – tet – OriT – FcpF – 3′). After cloning and *E. coli* transformation, the correctly assembled episome, pBR_Cas9-2A-sh ble, 2XBsaI-mRFP(AT) (Supplementary Figure S3A), was sequence verified.

Next, primers were designed to PCR amplify up to six sgRNA expression cassettes (cloning and construction described above) and to add *Bsa*I-restriction and cleavage sites to the 5′ and 3′ ends of the sRNAi promoter/terminator. GG cloning is an optimal assembly platform when cloning more than one sgRNA expression cassette. For inserting one sRNAi_sgRNA cassette, primers GG-gRNA1-F and sgRNA(GG)-2 were used. For two sgRNAs, primers GG-gRNA1-F and R were used for sgRNA #1 and primers GG-gRNA3-F and sgRNA(GG)-2 were used for sgRNA #2. Between the two sgRNAs was cloned a LacZ expression cassette, which, when induced with IPTG activates beta-galactidose that digests Xgal to produce blue *E. coli* colonies (Supplementary Figure S3C). The sgRNA expression cassette(s) and LacZ amplicons was mixed at double the molar concentration of the *Bsa*I-digested pBR_Cas9-2A-sh ble, 2XBsaI-mRFP(AT) vector and carried through the GG cloning reaction with *Bsa*I-HF and a T4-ligase (Supplementary Figure S1C). The final sgRNA-LacZ-sgRNA episomal plasmid map can be seen in Supplementary Figure S3B.

Detailed protocols for sgRNA cloning into the Cas9-2A-sh ble epsiome (named GG2 cloning) is available at protocols.io (doi: 10.17504/protocols.io.4acgsaw).

### *Phaeodactylum tricornutum* Genetic Transformations

#### Bacterial-Mediated Conjugation

**FIGURE 1:**
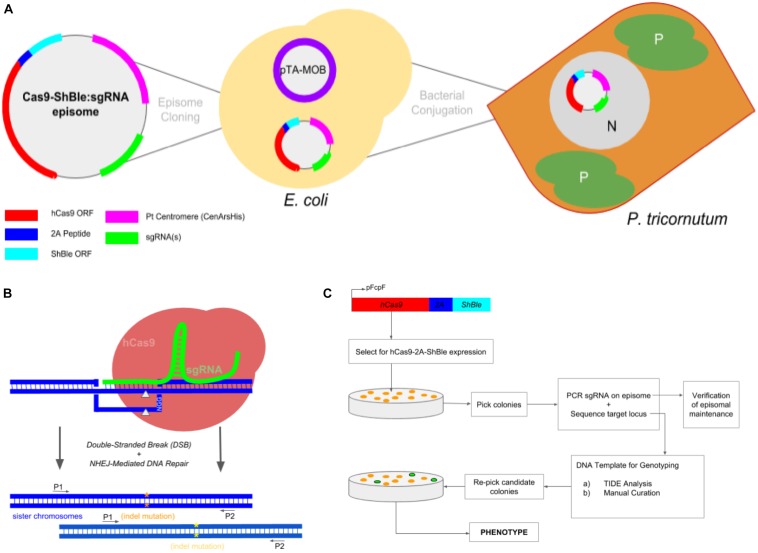
Experimental workflow to produce *Phaeodactylum* mutants via conjugation-based delivery of a CRISPR-Cas9 episome. **(A)** A cartoon schematic of Cas9-Sh ble:sgRNA episome cloning to *E. coli* cells harboring the pTA-MOB plasmid and followed by bacterial-conjugation transformation of the episome to *Phaeodactylum*. *Phaeodactylum* cells maintain and replicate the Cas9-Sh ble:sgRNA episome as their native chromosomes in the nucleus (N). The diatom plastids (P) and cytoplasm (C) are labeled. Here, Cas9 is transcriptionally fused to sh ble and can therefore be selected for after transformation with the antibiotic phleomycin. **(B)** Episomal expression of Cas9 and sgRNA(s) and target mutagenesis of a diploid organism. The sgRNA(s) guides Cas9 to genomic target(s), and then Cas9 induces a double-stranded break (gray arrows). *Phaeodactylum*, being a diploid eukaryote, will contain two distinct NHEJ-mediated mutations (orange and yellow asterisks) between the sister chromosomes. **(C)** Genotyping and cell line picking workflow after colony selection for Cas9-Sh ble. A “double picking” strategy was used by which cell lines were picked and target locus sequenced using two primers. After genotyping, the colony can be re-picked for further cell line analysis.

Bacterial-mediated conjugation was used to introduce CRISPR components to *Phaeodactylum*. Diner et al., 2016 provided detailed methodology for introducing episomal plasmids to *Phaeodactylum* in a high-throughput manner. For all transformations, 100 uL of dense *Phaeodactylum* (1e8 cells/mL) was plated on conjugation-based solid agar medium (NH4/NO3-ASW, 1% agar, 5% LB in NH_4_-supplemented ASW) in 6-well cell culture plates and incubated for 4 days under light (18:6) and at 18°C. Prior to transformation, pBR_Cas9-2A-sh ble, sRNAi-sgRNA episomes were transformed into recipient *E. coli* harboring the conjugation plasmid pTA-MOB (Karas et al., 2015; Diner et al., 2016) and selected on agar plates for both the cargo and conjugation plasmids (Figure 1). For a transformation control (positive for colonies not Cas9 cleavage), the episome pBR_Cas9-2A-sh ble, 2XBsaI_gRFP1 was used where Cas9 expression would still occur without an accompanying sgRNA. For a negative control, a similar episomal plasmid was built harboring the NAT gene that confers resistance to nourseothricin and not phleomycin. Under phleomycin selection, the transformed cells would die. Transformation efficiency for the delivery of Cas9-2A-sh ble:sgRNA episomes was 500–750 transformants per 1 × 10^8^ phleomycin-resistant cells.

The day before transformation, 4 random colonies from the selection plates were used to inoculate 3 mL of LB containing selection (Amp, Tet, Gent). After growing overnight, 200 uL of culture was used to inoculate 12.5 mL of LB. The culture was grown for 3–4 h, until an OD-600 nm of 0.8–1 was reached, pelleted, and suspended in 100uL of SOC media. Then, the *E. coli* was pipetted into a corresponding well containing *Phaeodactylum* and mixed either by a spreading loop or by rotating the 6-well plate such that the *E. coli* covered the entire lawn of *Phaeodactylum*. After *E. coli* was added to all wells and allowed to dry under a vacuum PCR hood, the 6-well plate was placed in the dark at 30°C for 90 min. The plate was then placed back at 18°C and exposed to light for 48 h.

Finally, each well was scraped using a sterile cell spreader, collected in a 2 mL Epitube then re-plated on 100 mm selection plates containing the appropriate selective media. The selection plates were then allowed to grow for 10–14 days or until colonies appeared. For the T7 Endonuclease I Assay reaction, half of the scraped *Phaeodactylum* cell lines (∼400 uL) was resuspended in 200 mL NH_4_-supplemented ASW media supplemented with phleomycin and chloramphenicol. The cells were then grown under light and at 18°C for 4 days, passed into 200 mL fresh media, grown for an additional 3 days, then finally pelleted and flash frozen.

Detailed protocols for bacterial-conjugation in *Phaeodactylum* are publicly available at protocols.io (doi: 10.17504/protocols.io.5pvg5n6).

#### Micro-Particle Bombardment Genetic Transformation

Micro-particle bombardment genetic transformation (Falciatore et al., 1999) was used to introduce CRISPR components to *Phaeodactylum*. First, *Phaeodactylum* was pelleted during exponential growth in liquid culture (NO_3_-supplemented ASW), suspended, and plated onto agar growth plates at a cell concentration of 3e8 cells/mL (400 uL). Next, 24 ug of DNA (8 ug each plasmid; FcpB-Cas9, sRNAi-gNR-B, pKO-NR) were hybridized to tungsten beads and introduced to the plated *Phaeodactylum* at high velocity using the PDS-1000/He Biolistic^®^ Particle Delivery System (Bio-Rad, Hercules, CA). The transformed *Phaeodactylum* plates were then allowed to recover for 48 h at 18°C and in constant darkness. After recovery, *Phaeodactylum* was re-plated on agar plates containing NH_4_-supplemented ASW with *phleomycin* to select for the presence of the pKO-NR plasmid. The selection plates were then grown under normal conditions for 21–28 day, or until *Phaeodactylum* colonies formed and were visible. Experimental controls were also performed where either FcpB-Cas9 and pKO-NR were co-transformed or pKO-NR was transformed alone.

### T7 Endonuclease I Assay for Determining Cas9 Mutagenesis Efficiency

**FIGURE 2:**
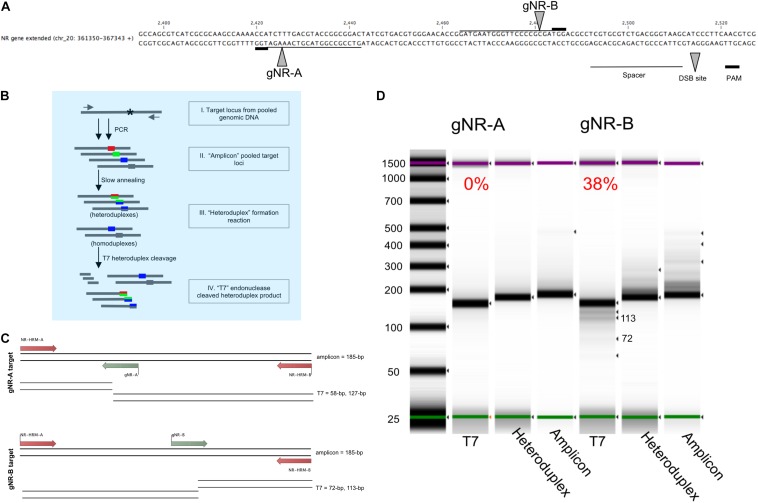
Pooled-diatom transformant T7 assay screen for *in vivo* Cas9 activity to compare the mutagenesis efficiency between two sgRNAs designed to target the *nitrate reductase* (Phatr3_J54983) gene. **(A)** Two sgRNAs were designed within the *Phaeodactylum* genomic region displayed. The spacer sequence (line), PAM site (bold line) and Cas9 cut site (grew arrow) are defined for sgRNA gNR-A and gNR-B. **(B)** The T7 heteroduplex assay was used to quantify the efficiency of *in vivo* Cas9 activity for each sgRNA for a population of pooled-*Phaeodactylum* transformants. This chart demonstrates how the T7 heteroduplex assay can produce and cleave mismatched amplicon products(I). The pool of target loci amplicons (II) is denatured and slowly annealed to form mismatched double-stranded DNA, or heteroduplexes (III). The heteroduplexes are then digested by T7 endonuclease I (New England Biolabs). Heteroduplexes are subsequently cleaved by T7 while homoduplexes remain full length. **(C)** The expected T7 cleavage pattern for gNR-A and gNR-B associated amplicons. The size of the “amplicon” input is 185-bp. For gNR-A, the expected “T7” products are ∼185-bp (homoduplex), 127-bp and 58-bp. For gNR-B, ∼185-bp (homoduplex), 113-bp and 72-bp. **(D)** The three products, “amplicon,” “heteroduplex,” and “T7” were size analyzed on a 4200 TapeStation System (Agilent, Santa Clara, CA, United States). The T7 cleavage efficiency is displayed above each “T7” lane in the gel that represents the mutagenesis efficiency of each sgRNA for all *Phaeodactylum* cell lines. Each band detected by the software is indicated by a black arrow. The expected “T7” 113-bp and 72-bp bands are labeled.

The T7 endonuclease I Assay (New England Biolabs) was used to quantify the *in vivo* cleavage efficiency for multiple clonal *Phaeodactylum* populations for each sgRNA target. Frozen cell pellets obtained following liquid selection (as described above) were slowly thawed on ice. Genomic DNA was then extracted from the pelleted cells using the Plant DNAzol Reagent (Thermo Fisher Scientific). The product-supplemented protocol was used; however, the pulverization step, the first step, was skipped because diatom pellets can be easily resuspended. Genomic DNA was then extracted from the cell pellet. 200 uL of genomic DNA at concentration ranging from 200 to 500 ng/uL was recovered. A total of 200 ng of genomic DNA was used as a PCR template to start the T7 endonuclease I assay. Six separate amplifications were performed (only 25X amplification cycles) and pooled prior to the T7 assay. For the *NR* locus, primers NR-gene-1 and NR-KO-2^∗^ were used. This amplification product was then re-amplified with nested primers NR-HRM-A and NR-HRM-B. The nested-amplicons were then PCR cleaned and concentrated and used as a DNA input for the heteroduplex hybridization. This input is referred to as “Amplicon” in Figure 2 and the hybridized heteroduplex is referred to as “Heteroduplex.” T7 endonuclease I was then added to the hybridized product to cleave all heteroduplex DNA. All reaction conditions and efficiency calculations (as shown in Figure 2) were supplied by New England Biolabs (protocol website^1^). Genome editing efficiency was determined using the Agilent 4200 TapeStation bioanalyzer and Agilent High-Sensitivity D1000 screen chip and assay. The fraction cleaved value was determined by examining the peak intensities of the high-sensitivity gel of the T7 product compared to the uncut Heteroduplex product. Fraction cleaved was then an input for the following equation to calculate the percentage of gene modification:

$$\%genemodification=100*\left(1-{\left(1-fractioncleaved\right)}^{1/2}\right).$$

### Genotyping *Phaeodactylum* Exconjugants

*Phaeodactylum* cells selected on solid media were grown for 10–14 day or until visible colonies formed. Colonies were picked with pipette tips directly into 5uL dilution buffer supplied with the Direct Plant PCR Kit (Thermo Fisher Scientific) and incubated at RT for 15 min. The buffered colony was then pipette mixed and 2uL were used as a DNA template for two PCR reactions. The first, common for all sgRNAs, used primers Ars_Seq and V2_insert_2 (Supplementary Figure S4A, black arrows) to amplify from the Pt_Centromere to and through the sgRNA expression cassette to check for correct episomal maintenance and sgRNA presence (Supplementary Figure S4B). For *nitrate reductase*, primers NR-gene-1 and NR-KO-2^∗^ were used to amplify a 641 bp region around the target loci for gNR-A and gNR-B. The Direct Plant PCR Kit was used for both PCR reactions for 40 cycles. The resulting PCR amplicons were used for genotyping.

### TIDE Sequencing Analysis

**FIGURE 3:**
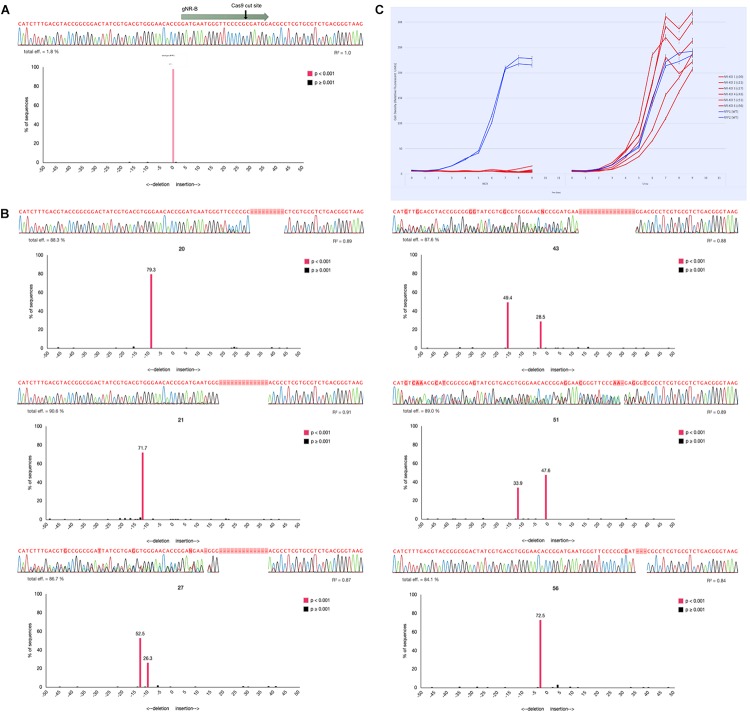
Genotyping NR-KO mutant cell lines by colony sequencing and TIDE sequence analysis. **(A)** The sequencing alignment paired with a TIDE chart associated with a wild-type *Phaeodactylum* NR sequence. The gNR-B spacer and PAM are represented by the gray arrow and the Cas9 cut site by the black arrow. Below is the sequencing read, the associated electropherogram, and the TIDE result chart. The TIDE chart is a bar plot where the *x*-axis represents the predicted indel mutation (0, wild-type, negative values, nucleotide deletion; positive values, nucleotide insertion) and the *y*-axis represents the percentage the specific mutation is of the entire sequence. A bar shaded pink indicates a *p*-value < 0.001 for that predicted indel. Here, there is one pink bar at position “0” (wild-type) that represents 97.1% of the entire sequence (labeled above the bar). **(B)** Six NR-KO mutant cell line genotypes were assessed the same as wild-type. The wild-type sequencing read was used as a reference input for TIDE analysis to compare potential mutant sequence genotypes. NR-KO mutant genotyping was performed for all colonies using both manual sequencing curation and TIDE analysis (Supplementary Table S2) and candidate NR-KO cell lines were chosen for phenotype analysis. Here, six NR-KO candidates and their associated genotyping results are shown. Cell lines 20, 21, and 56 display a single peak in the negative values that indicates a homozygous deletion genotype. Cell lines 27, 43, and 51 display two peaks in the negative values that indicate two deletions or a heterozygous deletion genotype. **(C)** The six candidate NR-KO cell lines were validated to have lost *nitrate reductase* function when grown in liquid medium supplemented with nitrate. Compared to growth on urea (left panel), the NR-KO mutant could not grow on nitrate (right panel). Two wild-type cell lines are blue and the six NR-KO cell lines are in red. All line were grown in technical triplicates.

TIDE (Tracking of Indels by DEconvolution, Brinkman et al., 2014) sequence analysis is an online-based software^2^ that analyzes raw sequencing data to find one or multiple indels mutations at a Cas9 target site. For the purposes of this study, TIDE was used to analyze indels resulting from Cas9 targeting of *nitrate reductase* in *Phaeodactylum* by sgRNAs gNR-A and gNR-B (Figure 3). An example of the TIDE analysis output for a wild-type sequence is shown in Figure 3A. 80 colonies for gNR-A and 64 colonies for gNR-B were picked directly into PCR mix (protocol described above). 200 ng of the PCR amplicons were then directly sequenced using two primers, NR-HRM-A and NR-gene-2, for paired-end reads (Figure 2A). *Phaeodactylum* transformed with the Cas9-2A-sh ble vector without an sgRNA was used as a control. TIDE analysis requires the input of (1) the nuclease species used (SpCas9), (2) the 20 nt spacer target, (3) an ABI (.abi) file for a reference sequence (control Cas9-2A-sh ble), and (4) an ABI (.abi) file for a sample sequence. The sample sequence is compared to the control sequence for significant differences between the sequence electropherograms. Certain criteria were identified and selected for when analyzing the TIDE output. First, a total efficiency value (in percentage) of 50% was chosen because it signified that the target locus was mutated at a high rate. Second, the *p*-value of each output peak must be <0.001. Third, only colonies with distinct genotypes, based on the criteria of only 1 or 2 predominant indels each with greater than 10% of the total sequencing reads, were selected as candidate mutant lines. Lastly, for the cell lines that passed each of these criteria, results were verified by conducting TIDE analysis of the paired-end read of the same sample. If both paired-end TIDE analyses pass the criteria and identified identical indels, the sample was chosen as a mutant cell line candidate. Each sequencing read was manually analyzed, individually, using the open-sourced TIDE web-tool.

### Nitrate Phenotype Assay for *Phaeodactylum* Mutant Cell Line Candidates

NR-KO *Phaeodactylum* cell lines were identified by TIDE and subjected to the NR phenotype assay where *Phaeodactylum* mutants of *nitrate reductase* are grown on media supplemented with nitrate and ammonium or urea, separately. The same NR assay was used for mutant cell lines produced via micro-particle bombardment. Mutants with a dysfunctional NR gene are unable to grow on nitrate media while proliferating on ammonium. First, all 40 colonies (for each sgRNA) were re-picked into liquid media supplemented with ammonium. The cultures (including four control cultures) were grown under normal conditions until mid-log phase and then passed into nitrate and ammonium media at a concentration of 1e4 cells/mL. Cell density was measured daily (2:00pm PST) for 4 days and growth between the two condition was compared. The cell lines that could grow on ammonium-supplemented ASW and could not grow on nitrate-supplemented ASW were considered NR mutant cell lines.

## Results

### CRISPR-Cas9 Mutagenesis Workflow in *Phaeodactylum* Using Selectable-Cas9 Episome

The general workflow from episomal exchange to *Phaeodactylum* colony genotyping is shown in Figure 1. The *Phaeodactylum* episome was built with a transcriptionally fused Cas9 and sh ble, an antibiotic selectable marker gene, expression cassette that allows Cas9 to be selected for after transformation with the eukaryote antibiotic phleomycin. Additionally, a red-fluorescent protein bacterial expression cassette (mRFP) was cloned into the episome with flanking *Bsa*I restriction enzyme sites that, when digested, leave unique 4-bp overhangs for golden-gate cloning of sgRNA constructs (Supplementary Figure S1). A hierarchical cloning strategy was used to build and clone one *Phaeodactylum* sgRNA expression cassette in two golden-gate assembly steps (Supplementary Figure S2). Negative selection against RFP was used for cloning an individual sgRNA expression cassette where negative *E. coli* colonies were visibly red. For cloning more than one sgRNA, a LacZ bacterial expression cassette was cloned with and between the sgRNAs that turns *E. coli* colonies with positively assembled episomes blue in the presence of IPTG and Xgal. The red-white-blue screening method helped expedite episome cloning in general (Supplementary Figure S3).

After construction and sequence verification, a Cas9-sh ble:sgRNA episome was transformed into electro-competent *E. coli* cells harboring a pta-MOB plasmid (Karas et al., 2015; Diner et al., 2016) that has been shown to be required for efficient conjugation exchange of the episome to *Phaeodactylum* (Figure 1A). The episome then resides in the *Phaeodactylum* nucleus, separate from the *Phaeodactylum* chromosomes as an artificial chromosome, where expression of Cas9 and sgRNA(s) occurs. Like many CRISPR/Cas9 gene targeting experiments in organisms with diploid genomes, the Cas9-sgRNA ribo-endonuclease complex is guided to both gene alleles where distinct NHEJ-mediated mutation may occur (Figure 1B). While a heterozygous genotype (two NHEJ-mediated mutations) was expected, a homozygous genotype was also expected where one mutated allele can serve as an HDR donor template to repair the second allele (HDR) or where micro-homology loci upstream and downstream of the cut site anneal following strand re-sectioning, referred to as MMEJ (Microhomology-Mediated End Joining; Wang and Xu, 2017).

Since both hetero- and homozygous mutations were expected, the genotyping workflow was conceived such that resulting colonies could be screened following selection for Cas9-2A-sh ble expression (Figure 1C). Colonies were first picked and added directly to PCR mix to amplify the sgRNA expression cassette on the episome harbored within *Phaeodactylum* (Supplementary Figure S4). Only colonies that contained a correctly sized amplicon were chosen for genotyping. While Cas9 expression was not verified for each individual colony, a Cas9-Venus (E-YFP) fusion was visualized by confocal microscopy to ensure localization in the *Phaeodactylum* nucleus (Supplementary Figure S5).

The methods described above were used for the following two applications: single-gene mutagenesis and two-gene mutagenesis.

### Single Gene Mutagenesis

Two sgRNAs were individually cloned into the Cas9-sh ble episome by golden-gate assembly (cloned episome product can be visualized in Supplementary Figure S2A). The sgRNAs were designed to mutate the *nitrate reductase* (NR) gene in *Phaeodactylum*. NR was picked as a genomic target because the function of NR has been well studied in *Phaeodactylum* and NR knockout cell mutants exhibit an easily screenable growth phenotype of cell death when supplemented with nitrate as a sole nitrogen source (McCarthy et al., 2017). The two sgRNAs were designed to target regions 55-bp apart so that the same amplification and sequencing primers could be used for genotyping efforts.

#### Comparative sgRNA-Effectiveness Assessment of Cas9-Episome Transformed *Phaeodactylum* Population

To quantify the mutagenesis efficiency for both sgRNAs population-wide, the T7 endonuclease assay was used (Figure 2). While the T7 assay has been typically used to genotype a clonal cell line, this method was useful to quantify the mutagenesis efficiency of all cells after transformation and phleomycin-selection of Cas9-2A-sh ble. This was done with the intention to identify sgRNAs that may produce low rates of mutagenesis and subsequently discard them. The sgRNAs were designed to target the NR locus 55-bp apart (Figure 2A). This allowed both targeted populations (one for gNR-A and one for gNR-B) to be subjected to the same T7 assay parameters (Figure 2B). The T7 enzyme cleaves mismatched nucleotides and should cut at Cas9 target loci with NHEJ-mediated mutations. The expected band sizes after T7 digestion was 127-bp and 58-bp for gNR-A and 113-bp and 72-bp for gNR-B (Figure 2C). Homoduplexes, annealed bands that perfectly match during “heteroduplex” formation (Figure 2B), was expected to be observed in the T7 migration gel because biallelic knockouts that arise from MMEJ-mediated repair may, in theory, anneal to each other and avoid T7 cleavage. It was observed that gNR-A insufficiently targeted the NR locus (0% mutagenesis efficiency) while gNR-B mutated the NR locus at 38% (Figure 2D).

By employing this method prior to colony genotyping, sgRNAs with low rates of mutagenesis throughout the population can be ignored which saves on reagent costs and labor. Henceforth, gNR-A was discarded from future targeting experiments due to its low mutagenesis efficiency compared to gNR-B.

#### Picking and Genotyping *Phaeodactylum*_NR-KO Mutants

TIDE (Tracking Indels by DEconvolution) sequence analysis was used to genotype *Phaeodactylum* cell lines (Figure 3). To do so, each sequence of the gNR-B target locus was first curated and analyzed manually. Next, one of two paired-end sequencing files were entered into the TIDE software and compared to a wild-type sequence for each colony. TIDE outputs a bar plot with predicted indel (insertion or deletion) mutations based on the inputted electropherogram compared to the wild-type. In Figure 3A, a wild-type NR sequence read is shown above the TIDE result plot that compares wild-type to wild-type. The TIDE plot displays one bar at *x*-axis position “zero” and with a *y*-axis value of 97.1% (displayed above bar). A pink colored bar also indicates the predicted indel was statistically significant (*p* < 0.001). This result indicates that there is a zero-nucleotide indel mutation at the predicted Cas9 cut site that comprises 97.1% of the total sequencing reads. Clearly, the TIDE plot validates the sequencing read as a wild-type genotype.

Figure 3B shows six NR knockout mutant genotypes for cell lines 20, 21, 27, 43, 52, and 56. Cell line 20, for example, appears to have a bi-allelic mutation that is homozygous because the TIDE chart shows one pink peak that corresponds to a deletion mutation of 9-bp. Cell line 43, for example, appears to have a bi-allelic mutation that is heterozygous because the TIDE charts shows two pink peaks corresponding to a deletion genotype of 3-bp (28.5%) and 15-bp (49.4%). All six NR-KO genotypes display either homozygous or heterozygous genotypes by their TIDE charts. They also lack any peak at the “zero” position that would indicate that there was a wild-type contamination. Furthermore, cell lines with TIDE charts with only 1 or 2 indel peaks are shown here; additional peaks would indicate that the cell lines were not clonal and need to be re-streaked to find a clonal mutant.

Manual sequencing curation and TIDE analysis was performed for 80 gNR-A colonies and 64 gNR-B colonies. 2/80 (2.5%) gNR-A colonies and 26/64 (40%) gNR-B colonies contained a Cas9-mediated mutation. Here, the Cas9-sgRNA mutagenesis efficiencies of 0% and 28% calculated using T7 (Figure 2) reflects the efficiencies observed using TIDE.

Lastly, the nitrate growth assay was used to correlate the TIDE genotyping efforts to the expected NR mutant phenotype. The six mutant cell lines identified using TIDE did not grow on nitrate media compared to growth on ammonium media (Figure 3C).

### Two-Gene Mutagenesis

**FIGURE 4:**
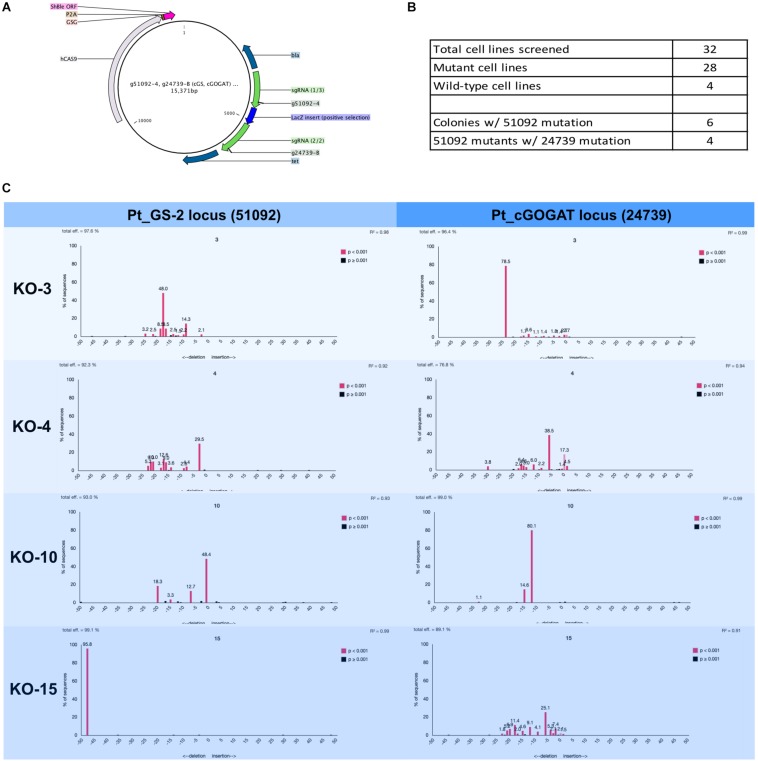
Two-gene mutagenesis of the genomic target glutamine synthetase 2 (GS-2, Phatr3_J51092) and the chloroplast localized glutamate synthase (cGOGAT, Phatr3_J24739). **(A)** The assembly Cas9-sh ble:sgRNA episome plasmid map with two sgRNA expression cassette (g51092-4 and g24739-B, green arrows). **(B)** A total of 28 phleomycin-resistant colonies and 4 wild-type cell lines were genotyped for mutations at both target loci. The screening was performed iteratively where all 32 cell lines (mutants and wild-types) were first screened for mutation at the g51092-4 target locus then candidate GS-2-KO cell lines were screened for mutation at the g24739-B locus. 6/28 cell lines exhibited Cas9 activity in 51092 and 4/6 GS-2 mutants exhibited Cas9 activity in 24739. The genotyping results for all cell lines was performed this way (Supplementary Tables S3, S4). **(C)** Four knock-out cell lines (3, 4, 10, and 15) and their associated TIDE charts show the diversity of mutagenesis at both loci.

The Cas9-2A-sh ble episome was assembled to harbor two sgRNA, one targeting glutamine synthetase 2 (GS-2, Gene ID: Phatr3_J51092) and one targeting a chloroplast-localized glutamate synthase (cGOGAT, Phatr3_J24739). The episome was assembled by golden-gate cloning and built to harbor both sgRNA expression cassette flanking a LacZ bacterial expression cassette (Figure 4A and Supplementary Figure S3).

#### TIDE Genotyping of *Phaeodactylum* Two-Gene Mutant Cell Lines

A total of 28 transformed *Phaeodactylum* colonies and 4 wild-type colonies were picked and genotyped using manual sequencing curation and TIDE sequencing analysis (Figure 4). The genotyping was performed successively. First, all 28 colonies were screened for mutation at the GS-2 target locus. Second, GS-2 KO candidate colonies were screened for mutation at the cGOGAT target locus. Therefore, genotyping information is not provided for all 28 colonies for the cGOGAT target locus. The genotyping results using manual curation and TIDE analysis for each colony is provided in Supplementary Table S3 (GS-2) and Supplementary Table S4 (cGOGAT).

A total of 6/28 (21%) colonies contained a Cas9-mediated mutation(s) at the GS-2 target locus and 4/6 GS-2 mutant candidates also contained Cas9-mediated mutations at the cGOGAT target locus (Figure 4B). Figure 4C shows the TIDE plots for each double-knock-out cell line (3, 4, 10, and 15) for both target loci (GS-2 on the left, cGOGAT on the right). Some mutations appear bi-allelic and homozygous, such as cell line 15 at the GS-2 locus (48-bp deletion) and cell line 3 at the cGOGAT locus (24-bp deletion). Only cell line 10 at locus cGOGAT displayed a bi-allelic, heterozygous mutation of a 15-bp deletion and 12-bp deletion. The remaining genotypes were not as clearly defined and have multiple (more than two) peaks in their TIDE chart and/or they contain a trace of a wild-type sequence (both examples are shown in cell line 4 at the cGOGAT locus).

### HDR-Mediated Gene Editing of NR in *Phaeodactylum* by Micro-Particle Bombardment

**FIGURE 5:**
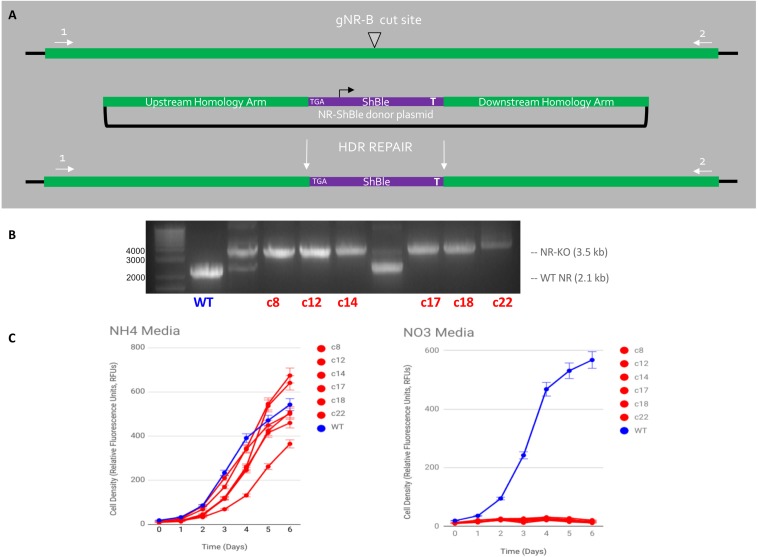
HDR-mediated CRISPR-Cas9 targeted insertion of Sh ble gene via micro-particle bombardment genetic transformation. **(A)** An *sh ble* expression cassette with an upstream premature STOP codon was precisely inserted at the gNR-B DSB site Cas9, gNR-B, and a NR-Sh ble donor plasmid were delivered to *P. tricornutum* cells on separate plasmids by micro-particle bombardment. The plasmids are presumably integrated within genomic DNA and the CRISPR components are expressed from *P. tricornutum* chromosomes. **(B)** An electrophoresis gel shows migrated amplicon from six mutants target loci compared to a wild-type NR locus. A single band, rather than two or more, indicated a bi-allelic HDR-mediated edit of the gNR-B target. **(C)** All six mutant cell lines could not grow on nitrate-supplemented media compared to wild-type but could grow on ammonium-supplemented media, a confirmation the predicted NR-KO mutant phenotype. All cell lines were grown in technical triplicates.

Six cell lines were produced via HDR-mediated gene editing of the NR gene in *Phaeodactylum* (Figure 5). It should be noted that, although attempted, HDR-mediated mutagenesis was not observed when the HDR-donor was encoded on the Cas9-sh ble episome (results not shown). All six cell lines exhibited the genotype (Figure 5B) and phenotype (Figure 5C) of having an HDR-mediated insertion of the sh ble expression cassette at the gNR-B target locus. An efficiency of 17% for producing a bi-allelic, HDR-mediated Pt_NR mutant was achieved.

## Discussion

CRISPR-Cas9 mutagenesis was performed in the model diatom *P. tricornutum* specifically to streamline the use of bacterial-conjugation transformation. First, the episomal delivery system for Cas9 was re-engineered so Cas9 could be selected for by phleomycin-antibiotic pressure. The 2A peptide transcriptionally fused Cas9 and the selective gene, sh ble, to ensure Cas9 could be selected for after conjugation. Previous attempts to deliver Cas9 in an expression cassette separate from the sh ble cassette resulted in low mutagenesis efficiency when colonies were not initially screened for a predicted phenotype, such as NR phenotype. Although not reported here, previously published experiments that delivered a non-selectable Cas9 via bacterial conjugation in *Phaeodactylum* resulted in mutagenesis efficiencies ranging from 25 to 33% using one sgRNA target (Sharma et al., 2018). Here, mutagenesis efficiencies ranged from 2 to 40% when targeting the nitrate reductase gene. Despite reporting a higher mutagenesis efficiency when using gNR-B, mutagenesis efficiencies cannot be directly compared when targeting two different genomic loci.

It has been observed that only 10% of transformed colonies contained a full Cas9 expression cassette, drastically lowering the mutagenesis efficiency (results not shown). It has been previously reported that the *Phaeodactylum* episome, naked, only retains its full plasmid sequence after transfer from *E. coli* to Phaeodactylum at 30% (Karas et al., 2015; Diner et al., 2016). Such a Cas9 episome design would not be ideal for targets without a previously known phenotype. The 2A peptide proved valuable in increasing the efficiency of Cas9 delivery to *Phaeodactylum* and in identifying mutant cell lines prior to checking for a phenotype response. Due to a transcriptional fusion to sh ble, it was presumed that the full Cas9 coding region was retained in all transformed Phaeodactylum transformants, though PCR amplification of the Cas9 ORF was not performed post-conjugation.

Although not thoroughly quantified, selection for Cas9 seems to alleviate previously reported problems of producing non-clonal *Phaeodactylum* that led to inevitable re-streaking, waiting for clonal cell lines to appear, and re-picking (Weyman et al., 2014). TIDE sequence analysis provided a predictive genotype for each cell line that can be interpreted as homozygous, heterozygous, or mixed genotype. For gNR-B, 26/64 cell lines exhibited Cas9 activity while 6 exhibited either a homo- or heterozygous genotype by TIDE analysis (Figure 3C), which can be interpreted to mean that those cell lines were clonal. However, 20/64 lines that passed the same efficiency criteria (in methods) contained more than 2 peaks in the TIDE chart or contained a trace wild-type sequence and therefore they were not considered clonal. Clonality was inherently tested for, also, when the NR-KO cell lines were subjected to a nitrate growth assay. If a wild-type cell contaminated the culture there would be growth on nitrate. Nevertheless, none of the six NR-KO cell lines grew on nitrate.

While TIDE sequence analysis is a useful tool to quickly screen raw sequencing reads for Cas9-induced indels. Here, TIDE was useful in identifying cell lines that contained targeted Cas9 activity and in inferring genotype, however, it is not recommended to use TIDE to confirm a cell lines genotype. Rather, the gold standard of sub-cloning the target locus into a TOPO vector followed by sequencing is recommended after TIDE analysis. It should be noted that TIDE analysis is complementary to CRISPR/Cas9 activity factor (CAF) analysis that was also designed to deconvolve diploid genotypes after CRISPR/Cas9 targeting (Stukenberg et al., 2018).

Re-streaking *Phaeodactylum* mutant is also recommended after TIDE analysis. The TIDE chart generated for gNR-B cell lines (Figure 3) suggest that the genotypes are either homozygous or heterozygous and that the cell lines are clonal. Nevertheless, TIDE is not the gold-standard for genotyping and assessment of clonality. As shown in Figure 4, not all TIDE plots indicate clonality and therefore re-streaking would be necessary to isolate double-knock-out mutant cell lines. All supplemental tables outline the genotyping efforts performed in this study. The last column for each table indicates the predicted genotype of the colony and suggests whether or not streaking the culture is recommended.

Lastly, the new Cas9 episome design decreased the time to produce and validate mutant cell lines. The time to produce visible colonies was 10–14 days, genotyping was 3 days, and phenotype screening was 10 days after re-picking. In total, the production of 6 *Phaeodactylum* mutants for the NR gene took 3–4 weeks compared to 4–8 weeks when using particle bombardment (Sharma et al., 2018). In a rapidly developing research landscape, optimization of time to produce mutant cell lines should not be a time-limiting step when there are questions regarding biological processes, ecological relevance, or biotechnology utility is an important goal.

## Data Availability Statement

The datasets generated for this study can be found in the MM Frontiers 2-27-29 Data Availability Document https://docs. google.com/spreadsheets/d/1snw_Jao9sdCDy2Y7cC3tP33cNzZ0 xTxOQTdHUEaARME/edit#gid=2069237253. Plasmid designed and mentioned in this manuscript will be made publicly available at the plasmid repository addgene.com.

## Author Contributions

MM was responsible for all of the molecular cloning, genetic transformations, genotyping assays, and writing. VB helped in plasmid and episome design, specifically for the conjugation protocols and implementation of the 2A peptide, and other intellectual contributions. PG performed the phenotype assays. MT designed golden gate primers for the episome. JM helped in daily tasks and manuscript writing. AA contributed to intellectual design and scope of the study. All authors contributed to the editing and feedback for this manuscript.

## Conflict of Interest

The authors declare that the research was conducted in the absence of any commercial or financial relationships that could be construed as a potential conflict of interest.

## Footnotes

1 https://www.neb.com/protocols/2014/08/11/determining-genome-targeting-efficiency-using-t7-endonuclease-i

2 https://tide.deskgen.com/

## Abbreviations

GA: Gibson assembly
GG: golden gate assembly
*Phaeodactylum*: *Phaeodactylum tricornutum;*
RT: room temperature.
