# Supplementary material for: Multiplexed Knockouts in the Model Diatom Phaeodactylum by Episomal Delivery of a Selectable Cas9
Source: Front Microbiol. 2020 Jan 28;11:5. doi: 10.3389/fmicb.2020.00005 (PMC6997545; doi:10.3389/fmicb.2020.00005)
Supplement: Supplementary file 1 [file Data_Sheet_1.pdf]

Supplemental Information. Sequences and a primer table

sgRNA expression components

1. sRNAi promoter sequence:

5' -
TCGACTTCTGCAGCTCTTCCAAATCGTACACGCCACTGGGCATTGAAGTGGACTCGCCTGCAGCATGAAA
ATTATCATAATAGCTGCAGGTTTCGATCGAGCCCGGACGCTTCTGTGCTTCTTCCAAAACCCAGCTCGCC
GTTAGACTTCGGCAAGCCGCATCGACGGCTTCCCGATCGCTTTCGGCCATGACTCGCTCGTGAACACACA
TATTCCGTCGACTGCTTAAACACAATGCTAGAGCCCCGCTTCCACCAGCTCCGCGACCGTCCGAGATCGG
TCCCATGGGAGGTTTTTGTAAATTGGCGGTATAGACCTTTCGAGGCTTCTTGCGATTGGGCGAGCCATTG
CTGGTAATGTGGGTGATGGCAGTGACGTTTACATCCCCGCTACTGCCCGCAACCGGAACGTTTTCAATAT
CCCCGTCAGTGTCCATTGGCAAGATGTTTTCTTCCTGGTGCCGTTGTAGTTCTTGCGACCGGTACGCTAA
AACTGTCGCGAGTTCTTCCATAACGTGATTCATTTCCGGAACGGTTCGAGTGCAATACACGAGCTTACCC
GCCGACGGATTCGCAAACTGGTAGGAAGTAATCAGCGATAGCAAACATACGGTTTTTCCCGTTCCCGTA
GGCATCTCCAGGAGACAGTGCCCACCTGCGTCCAGCGACTGTTTCAGTGCTCGCATGTACTGATGCTGCT
CAAGGTAGATGCGATCGTACGGAAAGAACACATCCAGGCCGTCCAAATCGAATCTCATTTTGACGCCTT
TTTCACACCTTCCGCTTATGCAAAGCTTTCAGGAATTGCCTATGAAGAGAACGGTCAATTCCTCATTGATG
ACTGACAAGACTTGTTTCTGTTTACGGTAATGAAGTCTCTCGCAATCCTTTTGCAATAGATCGATCGATCG
ACAGAATGGAAGCAACTTTTGATTACTTACTGTTACAGTTATTGTGTATAGGTATACTCTTTCTATTTATAT
ATTTGCAGAAAATCATAGTTTTG - 3'

underlined = TATA box (predicted)

2. sRNAi terminator sequence:

5' -
TTTTTGCCTTTTGACCGAAAGCTATCTTACTTACTACCTACCCAGCCACCGACAGCCTGTTTTCTATAGTAC
TGGAGCAAGACCAGAGTATAGAATTTGCTGCTGTTATAGCCGTT - 3'

3. sgRNA backbone sequence (redundant spacer):
5' -
NNNNNNNNNNNNNNNNNNNNGTTTTAGAGCTAGAAATAGCAAGTTAAAATAAGGCTAGTCCGTTAT
CAACTTGAAAAAGTGGCACCGAGTCGGTGCTTTTTT - 3'

4. 2X BbsI spacer insert:

5' -
GG**GTCTTC**GA**GAAGAC**CTGTTTTAGAGCTAGAAATAGCAAGTTAAAATAAGGCTAGTCCGTTATCAACT
TGAAAAAGTGGCACCGAGTCGGTGCTTTTTTT - 3'

Bold = BbsI-HF recognition site

5. 2X BBsI LacZ insert:

5’ -
TATATATTTGCAGAAAATCATAGGG**GTCTTC**GGCAGCTGGCACGACAGGTTTCCCGACTGGAAAGCGGG
CAGTGAGCGCAACGCAATTAATGTGAGTTAGCTCACTCATTAGGCACCCCAGGCTTTACACTTTATGCTT
CCGGCTCGTATGTTGTGTGGAATTGTGAGCGGATAACAATTTCACACAGGAAACAGCTATGACCATGAT
TACGCCAAGCTTGCATGCCTGCAGGTCGACTCTAGAGGATCCCCGGGTACCGAGCTCGAATTCACTGGC
CGTCGTTTTACAACGTCGTGACTGGGAAAACCCTGGCGTTACCCAACTTAATCGCCTTGCAGCACATCCC
CCTTTCGCCAGCTGGCGTAATAGCGAAGAGGCCCGCACCGATCGCCCTTCCCAACAGTTGCGCAGCCTG
AATGGCGAATGGCGCCTGATGCGGTATTTTCTCCTTACGCATCTGTGCGGTATTTCACACCGCATATGGT
GCACTCTCAGTACAATCTGCTCTGATGCCGCATAGTTAAGCCAGCCCCGACACCCGCCAACACCCGCTGA
CGCGCCCTGACGGGCTTGTCTGCTCCCGGCATCCGCTTACAGACAAGCTGTGACA**GAAGAC**CTGTTTTA
GAGCTAGAAATAGCAA - 3’

Bold = BbsI-HF recognition site

<u>CRISPR-Cas9 episome components</u>

GSG-P2A Sequence:
5’ -
GATGAATGGGTTCCCCGCGAGTTTTAGAGCTAGAAATAGCAAGTTAAAATAAGGCTAGTCCGTTATCAA
CTTGAAAAAGTGGCACCGAGTCGGTGCTTTTTT - 3’

<u>Primer List</u>

Episome Cloning Primers	
pBR322-Amp_1	CAATGATACCGCGgGAtCCAC
pBR322-Amp_2	GCGTGGaTCcCGCGGTATCATTG
Cas9-2A-ShBle_1	GTATCGATAATATTCTAGCTGAGGGTACCCATGGACTATAAGGACCACGA
Cas9-2A-ShBle_2	ctggcgacgtggaggagaaccctggacctggactccggcgcATGGCCAAGTTGACCAGTG
Cas9-2A-ShBle_3	AACGCGCGGGGAGAGGCGGTTTGCGTATTggtctcatcgaTTCGGGGAAATGTGCGCGGAACC
Cas9-2A-ShBle_6	TTCGGGTGGGCCTTTCTGCGTTTATAggtctcacgttAAGTGCCACCTGACGTCTAAGAAACC

Cas9-2A-ShBle_7	TTGTAGTCTCCGTCGTGGTCCTTATAGTCCATGGGTACCCTCAGCTAGAAT
Cas9-2A-ShBle_RFP-1	TTCCGCGCACATTTCCCCGAAtcgatgagaccAATACGCAAACCGCCTCTCC
Cas9-2A-ShBle_RFP-2	TTAGACGTCAGGTGGCACTTaacgtgagaccTATAAACGCAGAAAGGCCCACCCG
P2A-1	agcaggctgaagttagtagctccgcttcccgtctcgcgcgcCTTTTTCTTTTTTGCCTGG
P2A-2	tccaggtccagggttctcctccacgtcgccagcctgcttcagcaggctgaagttagtagc

sgRNA target loci	
gNR-A	*GTCCGCCGGTACGTCAAAGA***TGG**
gNR-B	*GATGAATGGGTTCCCCGCGA***TGG**
g51092	*GAGCCATCAAAGTTCCACTT****GGG***
g24739	*GATGACGTCAGCGTCAGCCT****TGG***

- PAM sequencing is bolded

Golden-gate insert primers for sgRNA spacer insert	
sRNAi_gNR-A_1	TACGgaagacAGTTTT*GTCCGCCGGTACGTCAAAGA*GTTTTAgtcttcGACA
sRNAi_gNR-A_2	TGTCgaagacTAAAAC*TCTTTGACGTACCGGCGGAC*AAAACTgtcttcCGTA
sRNAi_gNR-B_1	TACGgaagacAGTTTT*GATGAATGGGTTCCCCGCGA*GTTTTAgtcttcGACA
sRNAi_gNR-B_2	TGTCgaagacTAAAAC*TCGCGGGGAACCCATTCATC*AAAACTgtcttcCGTA
sRNAi_g51092_1	TACGgaagacAGATAGTTTTGAGCCATCAAAGTTCCACTTGTTTTAgtcttcGACA
sRNAi_g51092_2	TGTCgaagacTAAAACAAGTGGAACTTTGATGGCTCAAAACTATCTgtcttcCGTA
sRNAi_g24739_1	TACGgaagacAGATAGTTTTGATGACGTCAGCGTCAGCCTGTTTTAgtcttcGACA
sRNAi_g24739_2	TGTCgaagacTAAAACAGGCTGACGCTGACGTCATCAAAACTATCTgtcttcCGTA

Cloning and Reaction Primers	
GG-gRNA1-F	ATTTggtctcaTCGACTTCTGCAGCTCTTCCAAATC
GG-gRNA1-R	GCAcggtctcTACAGGAATTCGCTATAACAGCAGCAAATTCTATAC
GG-gRNA2-F	ATTTggtctcaCTGTCTTCTGCAGCTCTTCCAAATC
GG-gRNA2-R	GCACggtctctCTCAATCGATGCTATAACAGCAGCAAATT

GG-gRNA4-F	ATTTggtctcaAACCCTTCTGCAGCTCTTCCAAATC
LacZ-GG-1	ATTTggtctcaCTGTGGCAGCTGGCACGACAGGT
LacZ-GG-2	GCACggtctctCTCAATCGATTGTCACAGCTTGTCTGTAAGCGG
LacZ-GG-3	ATTTggtctcaTGAGGGCAGCTGGCACGACAGGT
LacZ-GG-4	GCACggtctctGGTTATCGATTGTCACAGCTTGTCTGTAAGCGG
sRNAi(GG)_2	GCACggtctctAACGGCTATAACAGCAGCAAATT
Amp-2	GAGAATTCTTGAAGACGAAGGGCC
ARS_seq	CGTGTAAGTTACAGGCAAGCG
V2_insert_2	AGCTTATCGATGATAAGCTGTC
NR-gene-1	GATGGATCAACTTAGTCTCCTCG
NR-gene-2	GCTACGATTTCGTCCATCGA
NR-KO-2*	ATTGTTTGACGGATCATGTT
NR-HRM-A	GAAGCTCGCGAAGGTGACACTCC
NR-HRM-B	TGCTTCAGAATCGCCAGCGGTG
NR-HRM-C	CCAGCGTCATCGCGCAA
NR-HRM-D	GTGGTTCGACGTTGAAGGGA
GSII_transcript_1	ATGAAATTAAACATTGCTGCTATTGCG
GSII_KO_Seq1	ACACGCACGCTTCCTGCCAA
GSII_KO_Seq4	CCTGTTGTCCGGGCATGA
GSII_KO_Seq5	CATGGATTCCGTAATGTGACG
24739_KO_1	ACAGCTGGAGATTAAAATGG
24739_KO_2	ATAACACAGCCTTCTGCA
24739_KO_3	GGTGTCACGCTGATATCACC
24739_KO_4	CCATCGACACGAAGCGTTAC
