# Supplementary figures and images for: Multiplexed Knockouts in the Model Diatom Phaeodactylum by Episomal Delivery of a Selectable Cas9
Source: Front Microbiol. 2020 Jan 28;11:5. doi: 10.3389/fmicb.2020.00005 (PMC6997545; doi:10.3389/fmicb.2020.00005)

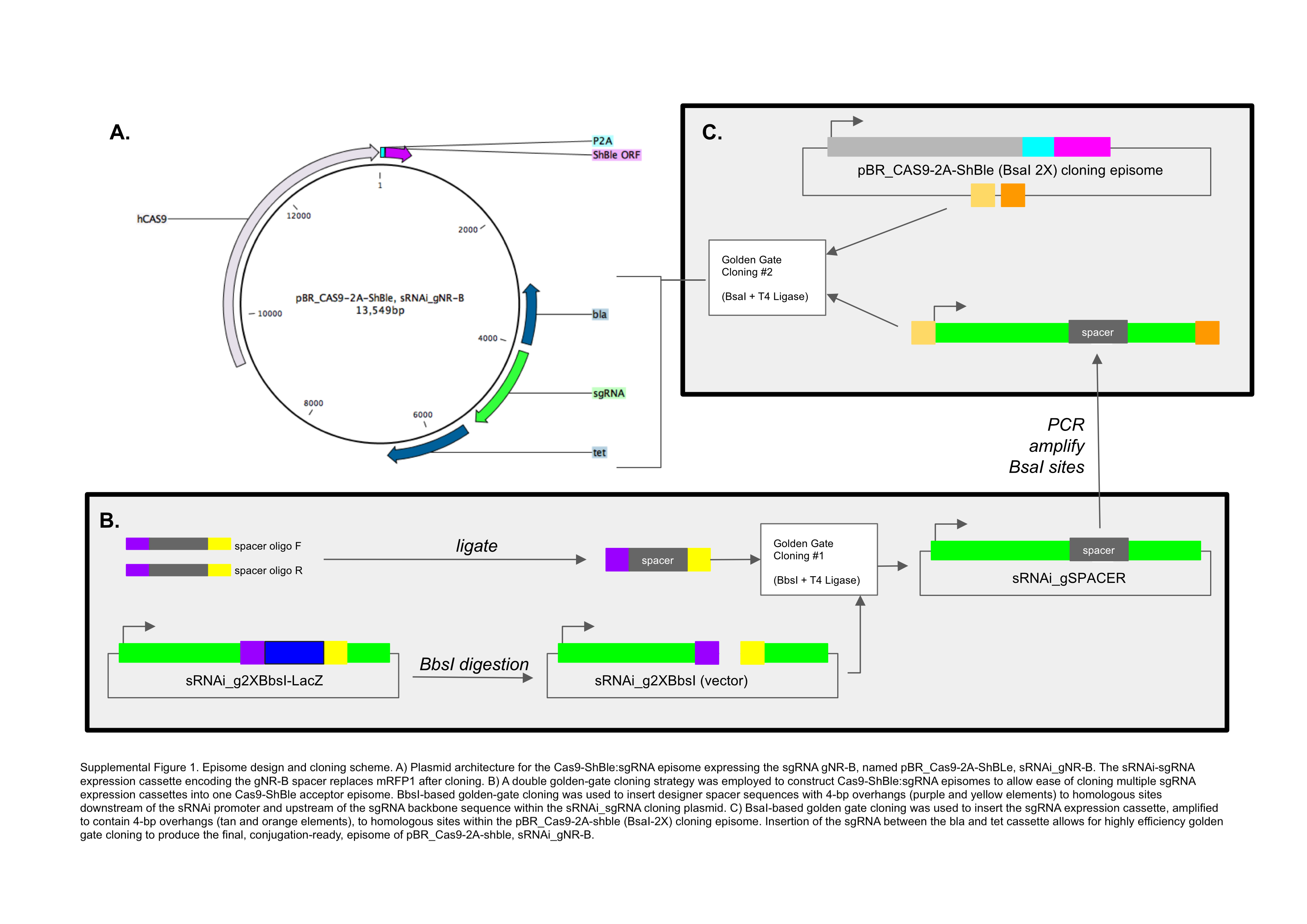

Supplement: Supplementary file 2 [file Image_1.jpeg]

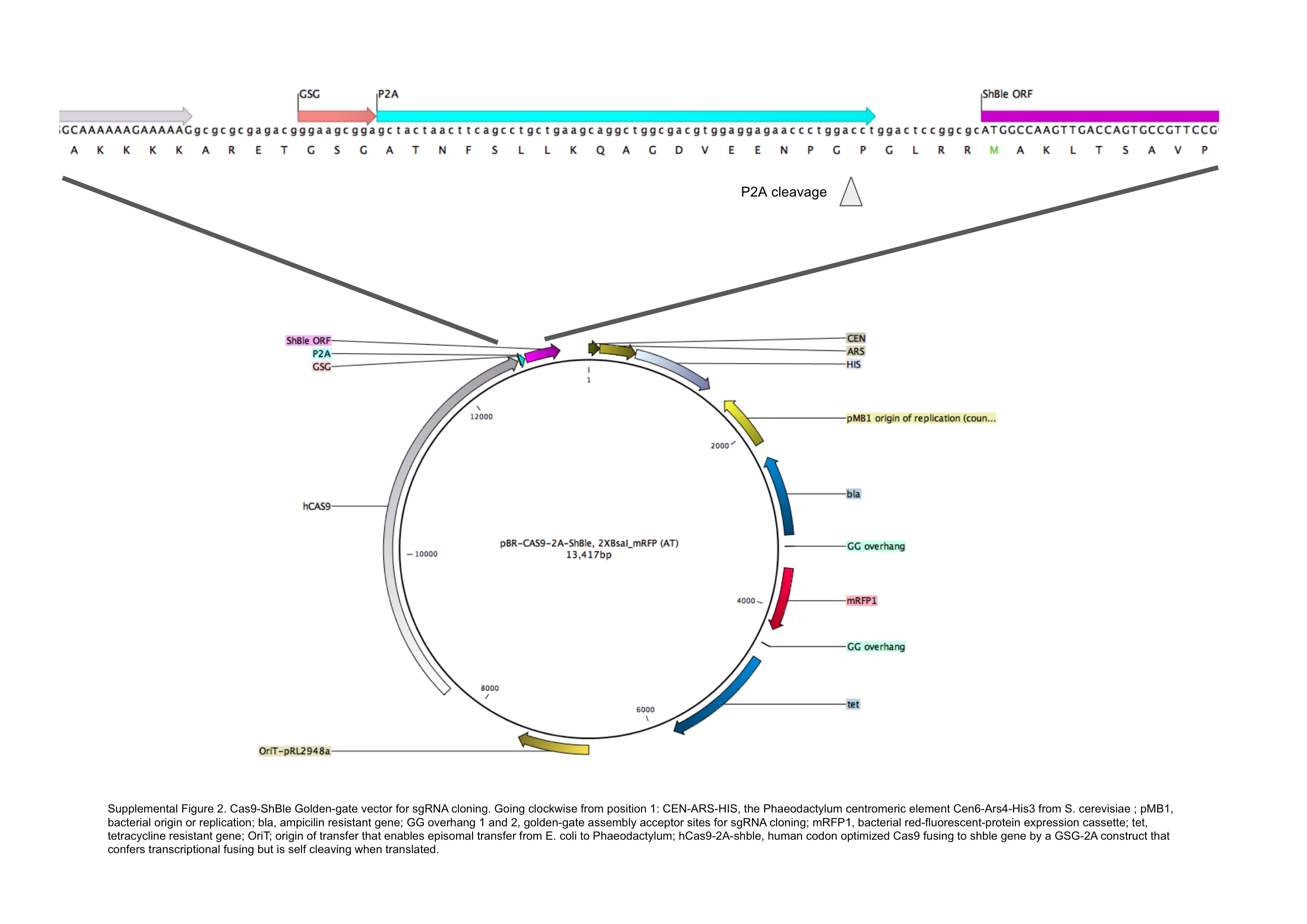

Supplement: Supplementary file 3 [file Image_2.jpeg]

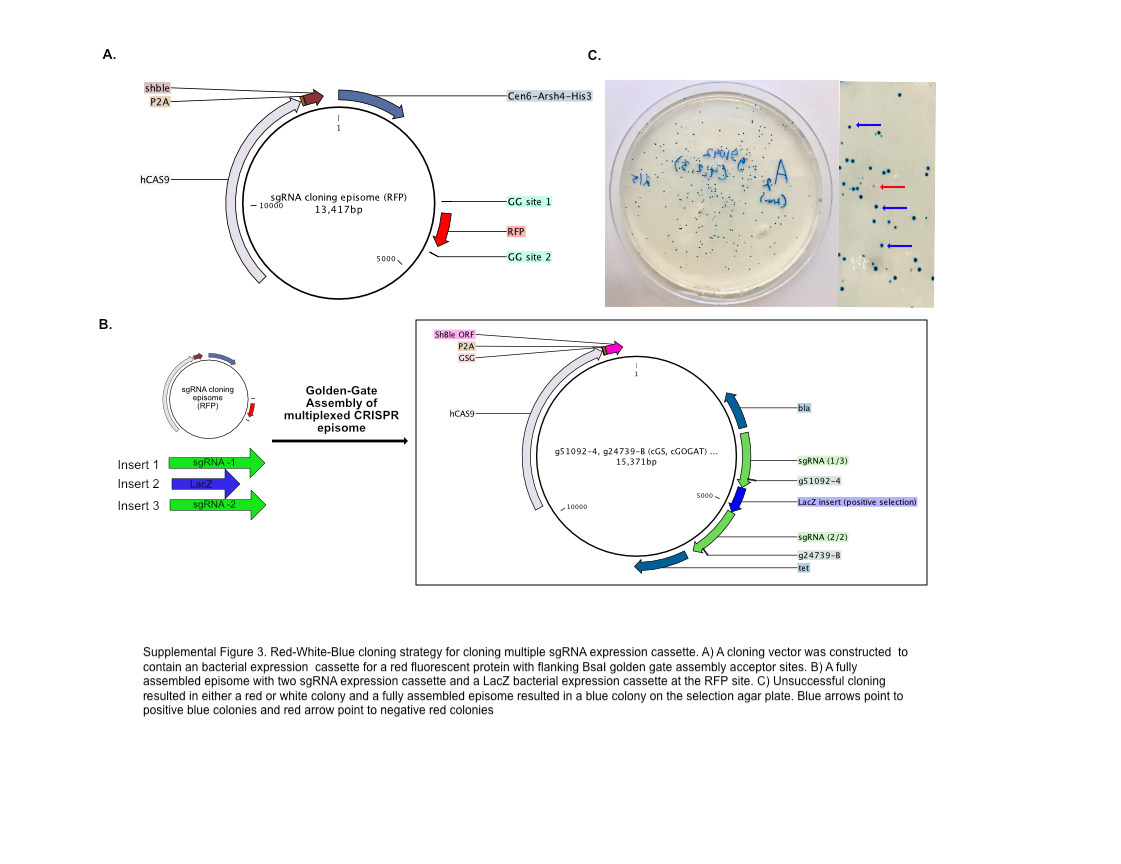

Supplement: Supplementary file 4 [file Image_3.jpeg]

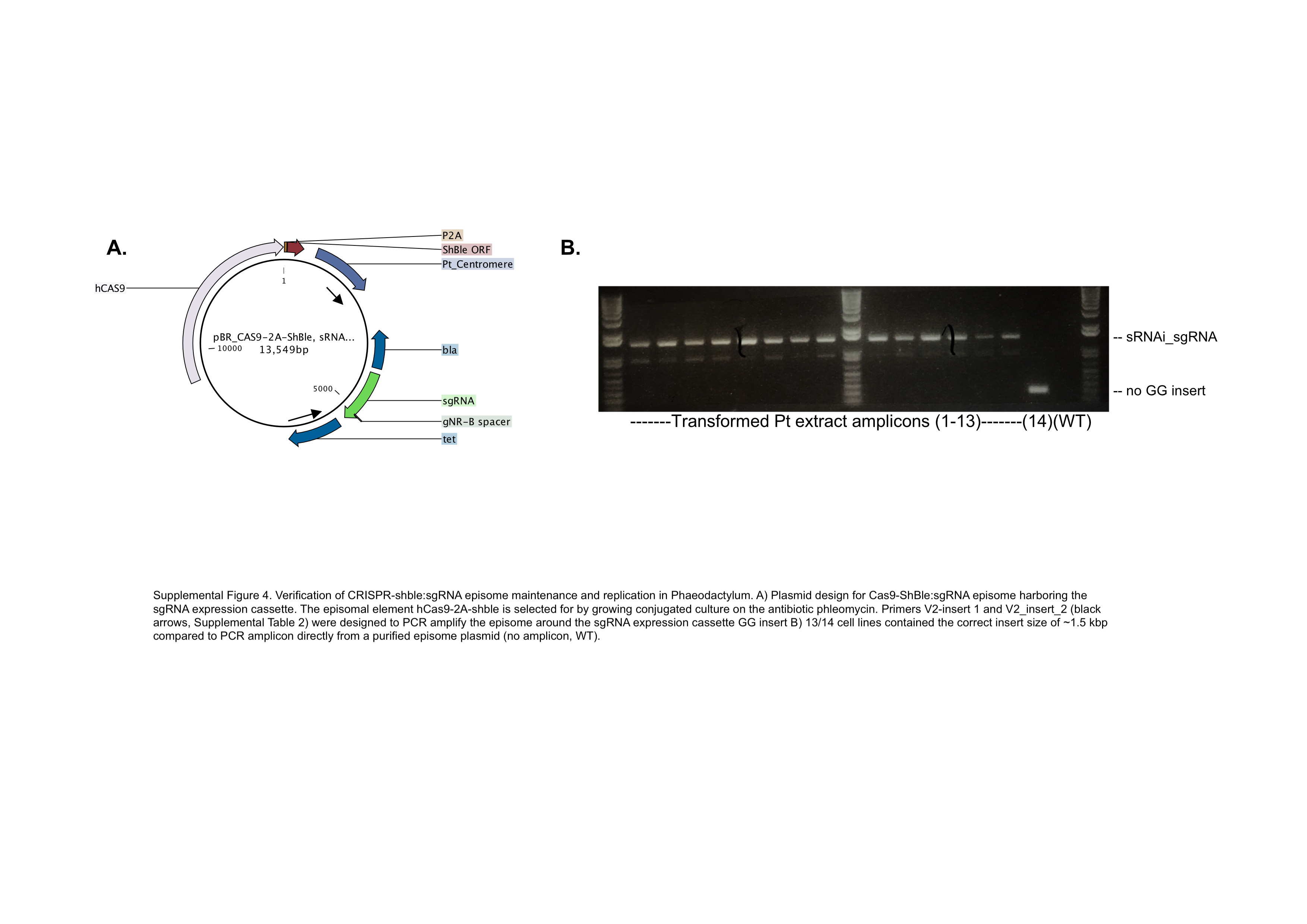

Supplement: Supplementary file 5 [file Image_4.jpeg]

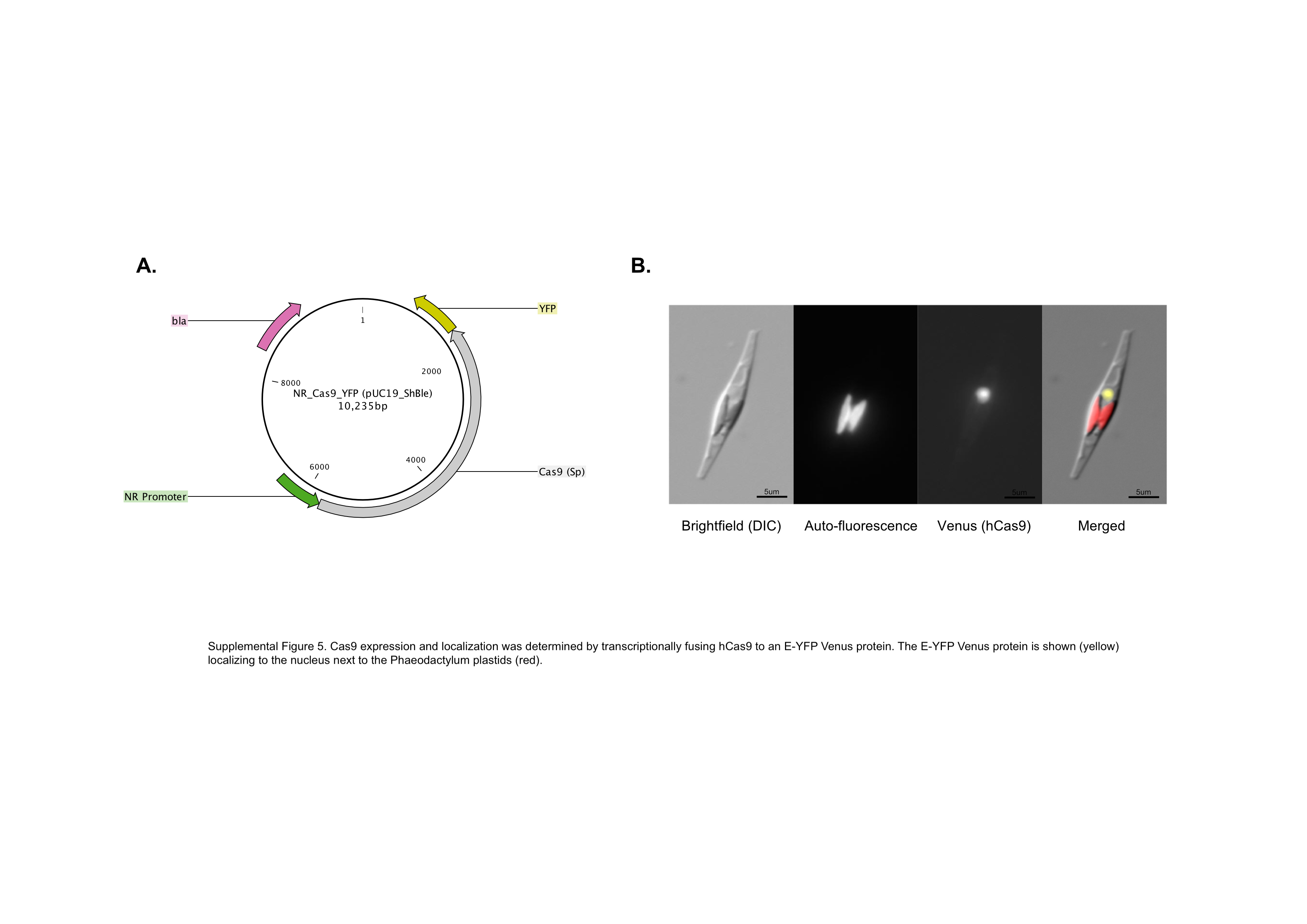

Supplement: Supplementary file 6 [file Image_5.jpeg]
