# Supplementary material for: Multiplexed Knockouts in the Model Diatom Phaeodactylum by Episomal Delivery of a Selectable Cas9
Source: Front Microbiol. 2020 Jan 28;11:5. doi: 10.3389/fmicb.2020.00005 (PMC6997545; doi:10.3389/fmicb.2020.00005)
Supplement: Supplementary file 7 [file Table_1.pdf]

Supplemental Table 1. gNR-A cell lines genotyping

		TIDE	
**Colony**	**Manual Curation**	**Predicted Mutation**	**Predicted Genotype**
**1**	wild-type	N/A	N/A
**2**	wild-type	N/A	N/A
**3**	wild-type	N/A	N/A
**4**	wild-type	N/A	N/A
**5**	wild-type	N/A	N/A
**6**	wild-type	N/A	N/A
**7**	wild-type	N/A	N/A
**8**	14-bp deletion	1-bp insertion (adenine addition)	Homozygous
**9**	wild-type	N/A	N/A
**10**	wild-type	N/A	N/A
**11**	wild-type	N/A	N/A
**12**	6-bp deletion	94.9% wild-type	wild-type
**13**	wild-type	N/A	N/A
**14**	wild-type	N/A	N/A
**15**	wild-type	N/A	N/A
**16**	mixed	95.5% wild-type	wild-type
**17**	mixed	95.0% wild-type	wild-type
**18**	mixed	91.1% wild-type	wild-type
**19**	wild-type	N/A	N/A
**20**	wild-type	N/A	N/A
**21**	wild-type	N/A	N/A
**22**	wild-type	N/A	N/A
**23**	wild-type	N/A	N/A
**24**	wild-type	N/A	N/A
**25**	wild-type	N/A	N/A
**26**	wild-type	N/A	N/A
**27**	wild-type	N/A	N/A
**28**	wild-type	N/A	N/A
**29**	wild-type	N/A	N/A
**30**	mixed	1-bp insertion (adenine addition) (6.6% wild-type)	Homozygous (streak)
**31**	wild-type	N/A	N/A
**32**	wild-type	N/A	N/A
**33**	wild-type	N/A	N/A
**34**	wild-type	N/A	N/A
**35**	wild-type	N/A	N/A
**36**	wild-type	N/A	N/A
**37**	mixed	1-bp insertion (adenine addition)	Homozygous
**38**	wild-type	N/A	N/A
**39**	wild-type	N/A	N/A
**40**	wild-type	N/A	N/A
**41**	wild-type	N/A	N/A
**42**	wild-type	N/A	N/A
**43**	wild-type	N/A	N/A
**44**	wild-type	N/A	N/A
**45**	wild-type	N/A	N/A
**46**	wild-type	N/A	N/A
**47**	wild-type	N/A	N/A
**48**	wild-type	N/A	N/A
**49**	wild-type	N/A	N/A
**50**	wild-type	N/A	N/A
**51**	wild-type	N/A	N/A
**52**	wild-type	N/A	N/A
**53**	wild-type	N/A	N/A
**54**	wild-type	N/A	N/A
**55**	mixed	mostly wild-type	N/A
**56**	wild-type	N/A	N/A
**57**	mixed	mostly wild-type	N/A
**58**	wild-type	N/A	N/A
**59**	wild-type	N/A	N/A
**60**	wild-type	N/A	N/A
**61**	wild-type	N/A	N/A
**62**	wild-type	N/A	N/A
**63**	wild-type	N/A	N/A
**64**	mixed	96.3% wild-type	wild-type
**65**	wild-type	N/A	N/A
**66**	wild-type	N/A	N/A
**67**	wild-type	N/A	N/A
**68**	wild-type	N/A	N/A
**69**	wild-type	N/A	N/A
**70**	mixed	12-bp deletion, 2-bp deletion, 1-bp deletion (25.4% wild-type)	mixed
**71**	wild-type	N/A	N/A
**72**	wild-type	N/A	N/A
**73**	wild-type	N/A	N/A
**74**	wild-type	N/A	N/A
**75**	wild-type	N/A	N/A
**76**	wild-type	N/A	N/A
**77**	wild-type	N/A	N/A
**78**	wild-type	N/A	N/A
**79**	wild-type	N/A	N/A
**80**	wild-type	N/A	N/A
**81 WT**	wild-type	N/A	N/A
**82 WT**	wild-type	N/A	N/A
**83 WT**	wild-type	N/A	N/A
**84 WT**	wild-type	N/A	N/A
**85 WT**	wild-type	N/A	N/A
**86 WT**	wild-type	N/A	N/A
**87 WT**	wild-type	N/A	N/A
**88 WT**	wild-type	N/A	N/A
