# Supplementary material for: Multiplexed Knockouts in the Model Diatom Phaeodactylum by Episomal Delivery of a Selectable Cas9
Source: Front Microbiol. 2020 Jan 28;11:5. doi: 10.3389/fmicb.2020.00005 (PMC6997545; doi:10.3389/fmicb.2020.00005)
Supplement: Supplementary file 8 [file Table_2.pdf]

Supplemental Table 2. gNR-B cell line genotyping

		TIDE	
**Colony**	**Manual Curation**	**Predicted Mutation**	**Predicted Genotype**
**1**	14-bp deletion	14-bp deletion, 9-bp deletion, 2-bp deletion	mixed (streak)
**2**	mixed	21-bp deletion, 11-bp deletion, 3-bp deletion	mixed (streak)
**3**	wild-type	N/A	N/A
**4**	mixed	2-bp insertion, 9-bp deletion	heterozygous
**5**	14-bp deletion	14-bp deletion, 9-bp deletion, 8-bp deletion	mixed (streak)
**6**	N/A	N/A	
**7**	N/A	N/A	
**8**	15-bp deletion	15-bp deletion, 12-bp deletion	heterozygous
**9**	N/A	N/A	N/A
**10**	mixed	42-bp deletion, 25-bp deletion, 11-bp deletion	low mutagenesis frequency
**11**	mixed	N/A	N/A
**12**	mixed	N/A	N/A
**13**	mixed	33-bp deletion, 2-bp deletion, 1-bp insertion	low mutagenesis frequency
**14**	N/A	N/A	N/A
**15**	N/A	N/A	N/A
**16**	mixed	18-bp deletion (28.3% wild-type)	mixed
**17**	11-bp deletion	11-bp deletion	Homozygous
**18**	N/A	N/A	N/A
**19**	N/A	N/A	N/A
**20**	8-bp deletion	9-bp deletion	Homozygous
**21**	13-bp deletion	12-bp deletion	Homozygous
**22**	mixed	N/A	N/A
**23**	9-bp deletion	11-bp deletion (5.5% wild-type)	mixed
**24**	64-bp deletion	deletion too large for TIDE analysis	N/A
**25**	wild-type	N/A	N/A
**26**	mixed	15-bp deletion, 14-bp deletion, 13-bp deletion	mixed (streak)
**27**	13-bp deletion	13-bp deletion, 10-bp deletion	Heterozygous
**28**	mixed	13-bp deletion, 9-bp deletion	mixed (streak)
**29**	5-bp deletion	6-bp deletion, 8-bp deletion	heterozygous
**30**	mixed	N/A	N/A
**31**	wild-type	N/A	N/A
**32**	8-bp deletion	8-bp deletion	Homozygous
**33**	mixed	mixed	N/A
**34**	wild-type	N/A	N/A
**35**	12-bp deletion	13-bp deletion, 14-bp deletion	heterozygous
**36**	mixed	9-bp deletion, 6-bp deletion, 4-bp deletion	mixed (streak)
**37**	18-bp deletion	18-bp deletion, 12-bp deletion	heterozygous (streak)
**38**	N/A	N/A	N/A
**39**	mixed	14-bp deletion, 12-bp deletion (28.3% wild-type)	mixed
**40**	14-bp deletion	14-bp deletion (5.8% wild-type)	mixed
**41**	N/A	N/A	N/A
**42**	10-bp deletion	12-bp deletion, 1-bp deletion	mixed (streak)
**43**	14-bp deletion	16-bp deletion, 3-bp deletion	heterozygous
**44**	22-bp deletion	22-bp deletion, 19-bp deletion	heterozygous
**45**	N/A	N/A	N/A
**46**	N/A	N/A	N/A
**47**	18-bp deletion	17-bp deletion	Homozygous (streak)
**48**	62-bp deletion	deletion too large for TIDE analysis	N/A
**49**	mixed	N/A	N/A
**50**	10-bp deletion	19-bp deletion, 16-bp deletion, 1-bp deletion	mixed (streak)
**51**	mixed	12-bp deletion, 1-bp deletion	heterozygous
**52**	mixed	9-bp deletion, 23-bp deletion	mixed (streak)
**53**	12-bp deletion	13-bp deletion, 12-bp deletion. 8-bp deletion	mixed (streak)
**54**	N/A	N/A	N/A
**55**	mixed	9-bp deletion (20.2% wild-type)	mixed
**56**	3-bp deletion	3-bp deletion	Homozygous
**57**	N/A	N/A	N/A
**58**	wild-type	N/A	N/A
**59**	15-bp deletion	16-bp deletion, 14-bp deletion (2.8% wild-type)	mixed
**60**	10-bp deletion	11-bp deletion, 12-bp deletion (2.1% wild-type)	mixed
**61**	mixed	25-bp deletion, 14-bp deletion, 1-bp deletion	mixed (streak)
**62**	mixed	N/A	N/A
**63**	N/A	N/A	N/A
**64**	mixed	N/A	N/A
**65 WT**	wild-type	N/A	N/A
**66 WT**	wild-type	N/A	N/A
**67 WT**	wild-type	N/A	N/A
**68 WT**	wild-type	N/A	N/A
