# Supplementary material for: Multiplexed Knockouts in the Model Diatom Phaeodactylum by Episomal Delivery of a Selectable Cas9
Source: Front Microbiol. 2020 Jan 28;11:5. doi: 10.3389/fmicb.2020.00005 (PMC6997545; doi:10.3389/fmicb.2020.00005)
Supplement: Supplementary file 9 [file Table_3.pdf]

Supplemental Table 3. 2X KO genotyping (g51092 target locus)

		TIDE	
**Colonies**	**Manual Curation**	**Predicted mutation**	**Predicted Genotype**
**1**	mixed	wild-type	N/A
**2**	wild-type	N/A	N/A
**3**	3-bp insertion	18-bp deletion, 9-bp deletion	Heterozygous (streak)
**4**	mixed	3-bp deletion, mixed	Heterozygous (streak)
**5**	mixed	18-bp deletion, 14-bp deletion	Heterozygous (streak)
**6**	mixed	wild-type	N/A
**7**	mixed	wild-type	N/A
**8**	mixed	2-bp deletion	low mutagenesis frequency
**9**	mixed	wild-type	N/A
**10**	mixed	1-bp deletion, 7-bp deletion, 20-bp deletion	mixed (streak)
**11**	mixed	wild-type	N/A
**12**	wild-type	N/A	N/A
**13**	wild-type	N/A	N/A
**14**	wild-type	N/A	N/A
**15**	47-bp deletion	48-bp deletion	Homozygous
**16**	mixed	2-bp deletion	low mutagenesis frequency
**17**	wild-type	N/A	N/A
**18**	mixed	2-bp insertion	low mutagenesis frequency
**19**	mixed	low mutagenesis frequency	N/A
**20**	mixed	wild-type	N/A
**21**	mixed	1-bp deletion (4.8% wild-type)	mixed (streak)
**22**	wild-type	N/A	N/A
**23**	mixed	2-bp insertion	low mutagenesis frequency
**24**	mixed	1-bp deletion, 4-bp deletion	mixed (streak)
**25**	mixed	wild-type	N/A
**26**	wild-type	N/A	N/A
**27**	mixed	1-bp deletion (7.1% wild-type)	low mutagenesis frequency
**28**	wild-type	N/A	N/A
**29 WT**	wild-type	N/A	N/A
**30 WT**	wild-type	N/A	N/A
**31 WT**	wild-type	N/A	N/A
**32 WT**	wild-type	N/A	N/A
