# Supplementary material for: Multiplexed Knockouts in the Model Diatom Phaeodactylum by Episomal Delivery of a Selectable Cas9
Source: Front Microbiol. 2020 Jan 28;11:5. doi: 10.3389/fmicb.2020.00005 (PMC6997545; doi:10.3389/fmicb.2020.00005)
Supplement: Supplementary file 10 [file Table_4.pdf]

		TIDE	
**Colony**	**Manual Curation**	**Predicted Mutation**	**Predicted Genotype**
**3**	24-bp deletion	24-bp deletion (2.7% wild-type)	Homozygous (streak)
**4**	6-bp deletion	6-bp deletion (17.3% wild-type)	Homozygous (streak)
**5**	wild-type	N/A	N/A
**10**	12-bp deletion	12-bp deletion, 15-bp deletion	Heterozygous
**15**	6-bp deletion	6-bp deletion, 18-bp deletion (2.1% wild-type)	mixed
**21**	6-bp deletion, mixed	low mutagenesis frequency	N/A
**WT1**	wild-type	N/A	N/A
**WT2**	wild-type	N/A	N/A

Supplemental Table 4. 2X KO genotyping (g24739 target locus)
